# Kinetics of substrate utilization and bacterial growth of crude oil degraded by *Pseudomonas aeruginosa*

**DOI:** 10.1186/s40201-015-0221-z

**Published:** 2015-09-24

**Authors:** Amirreza Talaiekhozani, Nematollah Jafarzadeh, Mohamad Ali Fulazzaky, Mohammad Reza Talaie, Masoud Beheshti

**Affiliations:** Department of Civil and Environmental Engineering, Jami Institute of Technology, Isfahan, Iran; Centre for Environmental Sustainability and Water Security, Research Institute for Sustainable Environment, Universiti Teknologi Malaysia, 81310 UTM Skudai, Bahru, Johor Malaysia; Department of Environmental Health, School of Health, Jondishapour University of Medical Science, Ahwaz, Iran; Faculty of Civil Engineering, Universiti Teknologi Malaysia, Bahru, Johor Malaysia; Department of Chemical and Environmental Engineering, Faculty of Engineering, University of Nottingham Malaysia Campus, Kuala Lumpur, Malaysia; Department of Chemical Engineering, Isfahan University, Isfahan, Iran

**Keywords:** Bacterial growth, Crude oil, Kinetic model, *Pseudomonas aeruginosa*, Substrate utilization

## Abstract

Pollution associated with crude oil (CO) extraction degrades the quality of waters, threatens drinking water sources and may ham air quality. The systems biology approach aims at learning the kinetics of substrate utilization and bacterial growth for a biological process for which very limited knowledge is available. This study uses the *Pseudomonas aeruginosa* to degrade CO and determines the kinetic parameters of substrate utilization and bacterial growth modeled from a completely mixed batch reactor. The ability of *Pseudomonas aeruginosa* can remove 91 % of the total petroleum hydrocarbons and 83 % of the aromatic compounds from oily environment. The value *k* of 9.31 g of substrate g^−1^ of microorganism d^−1^ could be far higher than the value *k* obtained for petrochemical wastewater treatment and that for municipal wastewater treatment. The production of new cells of using CO as the sole carbon and energy source can exceed 2^3^ of the existing cells per day. The kinetic parameters are verified to contribute to improving the biological removal of CO from oily environment.

## Introduction

The creation and evolution of technology for oil and gas industry can increase the capacity of oil and gas production [1, 2]. However, the operation of oil and gas refineries may negatively affect the environmental quality, including air and water pollution [3]. The increase in oil and gas production can have benefits of the government’s budget for the oil-producing countries but can also cause an increase in the release of pollutants into the environment. The chemicals in oil that are of most concern to humans are called such as total petroleum hydrocarbons (TPH) and aromatic compounds (AC) [4]. All of these chemicals may be released as water and air pollutants during the refining process. The petroleum refining processes of onshore/ offshore drilling and production having direct TPH and AC emission sources may pollute the waters. In most reservoir traps, the formation water pushes the oil residues from the oil reservoirs resulting in a mixture of oil and water that might be collected during the oil extraction process, and then the water phase can be separated from the crude oil (CO) by special techniques. The water contains varying amounts of TPH and AC and needs to be treated by an appropriate method to ensure the treated water quality to match stringent standards. The oily wastewater, also known as the produced water, is brought to the surface from underground formations during the oil and gas production; it can be found in many oil-producing countries [5]. The potential of oilfield produced water to be a source of fresh water and the increasing environmental concerns to stringent legislations on the wastewater released into the environment have made oilfield produced water management a significant challenge of the oil and gas business [6]. Having observed that the dissolved toxic hydrocarbons like TPH and AC commonly have a higher impact on the environment [7], they do not break down into harmless carbon dioxide (CO_2_) and water. Once the characteristics of the oily wastewater have been understood, the basic criteria for the selection of a definite separation technology can be proposed. The possibility of oily wastewater treated by physical, chemical and biological methods is an important process to ensure that the treatment system may be applied fairly. The use of locally isolated aerobic biosurfactant-producing bacteria has been researching more cost-efficient biological treatment methods, which are carried out with a minimum waste production and energy consumption [8–10]. The use of bacteria, yeast and fungi, which can grow using CO as the sole carbon source, has been reported with proper investigation of the hydrocarbons biodegradation cause [11–13].

Biological treatment is the most economic and eco-friendly process due to such a treatment offers least running cost, no requirement for hazardous chemicals and very low sludge production. The use of biosurfactant-producing bacteria is one way of removing many of the contaminants from many contaminated water, including TPH and AC from oily wastewater. Bioremediation is the promising technology for the treatment of oily wastewater [14–21]. Such a cost-effective technology of the mineralization may lead to complete breakdown of organic substances with the end products being CO_2_, H_2_O and nutrients [22]. The TPH in wastewater can be removed in 7 days of using the locally isolated oil-degrading bacteria with higher than 80 % efficiency [5]. Using a large-scale activated sludge to treat oily wastewater can remove 98 % of the TPH [23]. The presence of nutrients, especially nitrogen and phosphorus, in wastewater effluents and their impacts on the biodegradation of TPH are of major concern for biological wastewater treatment process [24]. The presence of rhamnolipids produced by *Pseudomonas aeruginosa* can play an important role in initiating the biodegradation of CO [25]. The effects of fuel concentration (diesel and gasoline), nitrogen concentration and culture type on the biodegradation of synthetic effluent have been studied to show that the potential of biodegradability can remove 90 % of the TPH over a process period of 49 days [21]. However, the systems biology approach to determine the kinetics of substrate utilization and bacterial growth still needs to be understood. The objectives of this study are: (1) to identify and select a suitable bacterial strain for degrading CO in a completely mixed batch reactor (CMBR) and (2) to verify the applicability of the kinetic models for the removal of CO from oily environment using the *Pseudomonas aeruginosa* as oil-degrading bacteria.

### Kinetic models

The kinetic models could allow defining the optimal conditions for the degradation of organic matter with bacterial activity from an oily environment [26]. The rate of microbial growth in an aqueous environment would be related to the concentration of a limiting organic matter. Many mathematical models of different levels of complexity have been developed to describe the growth of microorganisms. In this study, the use of the Monod equation could be useful to compute the rate of substrate utilization related to the specific growth rate [27], such that:

1 $$ - {r}_{su}=\frac{k \times X \times {S}_0}{K_s + {S}_0}=\frac{S_0 - S}{\theta } $$

where, *r*_su_ is the substrate utilization rate (in mg m^−3^ d^−1^), *k* is the maximum specific substrate utilization rate (in g of substrate g^−1^ of microorganisms d^−1^), *S*_o_ is the concentration of the limiting substrate for growth (in g m^−3^), *X* is the microorganism concentration (in g m^−3^), *K*_s_ is the half-velocity constant (in g m^−3^), *θ* is the residence time (in d), and *S* is the rest of the substrate concentration (in g m^−3^).

Rearranging Eq. (1) in the form of linear equation yields:

2 $$ \frac{X \times \theta }{S_0-S}=\frac{K_s}{k}\times \frac{1}{S_0}+\frac{1}{k} $$

It is clear that Eq. (2) is analogous to the linear equation [28, 29]: *Q* = *a × P* + *b*, where *a* is defined as slope and *b* is interception of curve $\frac{X \times \theta }{S_0-S}$ versus $\frac{1}{S_0}$, *Q* is $\frac{X \times \theta }{S_0-S}$ and *P* is $\frac{1}{S_0}$.

The residence time of water and free-living bacteria could be the important factor in utilizing the limiting substrate for growth, relative to bacterial growth rate, for such an endogenous decay to occur. Thus, the inverse of the residence time can be defined as

3 $$ \frac{1}{\theta }=-\frac{Y\times {r}_{su}}{X}-{k}_d $$

where, *θ* is the residence time (in d), *Y* is the synthesis yield coefficient (in g of biomass g^−1^ of substrate), *r*_su_ is the substrate utilization rate (in mg m^−3^ d^−1^), *X* is the microorganism concentration (in g m^−3^), and *k*_d_ is the endogenous decay coefficient (in g of microorganisms g^−1^ of biomass d^−1^).

When replacing –*r*_su_ with $\frac{S_0 - S}{\theta }$ in Eq. (3) yields a linear equation of:

4 $$ \frac{1}{\theta }=\frac{Y\times \left({S}_o-S\right)}{\theta \times X}-{k}_d $$

where, *θ* is the residence time (in d), *Y* is the synthesis yield coefficient (in g of biomass g^−1^ of substrate), *S*_o_ is the concentration of the limiting substrate for growth (in g m^−3^), *S* is the rest of the substrate concentration (in g m^−3^), *X* is the microorganism concentration (in g m^−3^), and *k*_d_ is the endogenous decay coefficient (in g of microorganisms g^−1^ of biomass d^−1^).

It is recognized that Eq. (4) is the linear equation: *J* = *Y × I* + *k*_d_, where *Y* is defined as slope and *k*_d_ is interception of curve $\frac{1}{\theta }$ versus $\frac{S_0-S\ }{X \times \theta }$, *J* is $\frac{1}{\theta }$ and *I* is $\frac{S_0-S\ }{X \times \theta }$.

The maximum specific growth rate of the bacteria is thus related to the maximum specific substrate utilization rate as follows [27]:

5 $$ {\mu}_{max}=k\times Y $$

where, *μ*_*max*_ is the maximum specific bacterial growth rate (in g of new cells g^−1^ of cells d^−1^), *k* is the maximum specific substrate utilization rate (in g of substrate g^−1^ of microorganisms d^−1^), *Y* is the synthesis yield coefficient (in g of biomass g^−1^ of substrate).

## Materials and methods

### Sampling procedure

It could be that the requirements for oil-degrading bacteria may rest largely on the petroleum-contaminated soils for several decades. Therefore, for the purpose of this work, one sample was taken from each location of oil-contaminated soils and effluents, i.e., (1) the soil around a gas station in Ahwaz city, (2) the soil around the Tehran oil refinery, (3) the outlet effluent of Tehran refinery wastewater treatment plant, and (4) the sanitary wastewater treatment plants of Tehran’s refinery. To do an assay, a 250 g of soil sample dispensed into 2 L sterile glass bottle was taken from each oil-contaminated soil location at 5 cm depth and a 1 L of wastewater sample filled into 1 L sterile plastic bottle was taken from each oil-contaminated effluent location at 80 cm depth. All the samples should be maintained at a safe temperature of less than 4 °C in a cold box during transportation to the laboratory.

### Cultivation, screening and activation of the oil-degrading bacteria

In this study, a 5 g of the soil sample was put into 500 mL of distilled water containing of 9 g L^−1^ NaCl in an Erlenmeyer flask, mixed, and shaken at 160 rpm for 30 min to have a suspension of bacteria in the liquid phase. Then 1 mL of the suspension bacteria was transferred to a 250 mL Erlenmeyer flask containing 100 mL of the nutrient solution (NS) and 1 mL of the CO as the sole carbon source and shaken at 160 rpm and incubated at 30 °C for 30 min. Notes that the NS consists of water containing a sufficient minerals i.e., 1 g L^−1^ NaNO_3_, 0.1 g L^−1^ MgSO_4_.7H_2_O, 0.01 g L^−1^ CaCl_2_, 0.001 g L^−1^ FeSO_4_, 0.5 g L^−1^ K_2_HPO_4_ and 0.5 g L^−1^ KH_2_PO_4_ and has a pH of 7.2. For the cultivation of bacteria, 1 mL of the incubated sample was used to enrich microbes degrading oil in a 250 ml Erlenmeyer flask containing 100 mL NS and 1 mL CO and shaken again at 160 rpm and 30 °C for 72 h. After that, 0.1 mL of the solution containing oil-degrading bacteria was selected streaked on a new NS agar plate and incubated at 30 °C for 48 h, and this step was repeated until pure cultures were obtained. Note that the bacterial colonies with different characteristics of color and size might grow on the NS agar. The pure cultures obtained were maintained on the NS agar slants at 4 °C and must be transferred to the new NS agar slants regularly every four months. Then the nutrients broth (NB) was used as the medium for the activation of oil-degrading bacteria. A large enough portion of the microbial growth from the slant culture was transferred to a 500-mL Erlenmeyer flask containing 100 mL of NB and shaken at 160 rpm and 30 °C and, after lasted for 24 h, it was placed in an approved device and spun at 3000 rpm in a centrifuge for 15 min and washed with sterile physiological saline solution to remove any contaminants. The bacteria were then suspended in physiological saline and could be ready for the future use.

### Measurement of crude oil degradation

A small portion of oil-degrading bacteria was inoculated into a 500 mL Erlenmeyer flask containing 100 mL of NS. Then 1 mL of CO as the sole carbon source was added to the Erlenmeyer flask and was kept on the shaking incubator at 30 °C and 160 rpm for 10 days, and then the amount of residual CO was measured after extraction of oil from the medium. Such experiment was repeated with different concentrations of CO. For the purpose of the study of kinetics, five 250 mL Erlenmeyer flasks (numbered 1, 2, 3, 4 and 5) containing 100 mL NS were used to measure the kinetics of substrate utilization and bacterial growth. After that, 1 ml of CO and 0.1 ml of the bacterial solution were aseptically added to each Erlenmeyer flask and incubated at 30 °C with shaking at 160 rpm. The rests of CO concentration in the Erlenmeyer 1, 2, 3, 4 and 5 were measured over a four days period after 12, 24, 40, 48 and 92 h exposure period, respectively. The data obtained from this data collection technique were used for the calculation and comparison of the kinetic parameters of the substrate utilization and bacterial growth.

### Analytical methods

A rapid spectrophotometric method based on a single optical density measurement was used to quantify biomass [30] and allowed the determination of biomass concentrations without the need for separation of the biomass from a solution. To measure the rest of CO in the solution, the pH was adjusted to 2 with 0.2 M HCl. Then the residual CO was extracted with 25 mL of carbon tetrachloride (CCl_4_) and then the sample was allowed to equilibrate for 15 min. After that, 1 mL of the lower organic phase was transferred into a clean 100 mL volumetric flask and made up to 100 mL with CCl_4_ and then the sample was centrifuged at 3000 rpm for 15 min in order to remove suspended particles and other substances that may interfere with the UV absorption measurement. The concentrations of TPH were determined using the UV spectrometer at 400 nm at which the maximum absorbance for a mixture of crude oil/CCl_4_ occurs. The emulsifying activity of biosurfactants produced by each bacterial strain was measured by emulsification index (E_24_) as described by Cooper and Goldenberg [31]. A 6 mL of the culture supernatant was mixed with 6 mL of kerosene in a tube test. The mixture was vortexed at high speed for 2 min and lasted subsequently for 24 h before the measurement of E_24_. The value of E_24_ was determined from the ratio of the thickness of the emulsion layer to the total thickness of the mixture and then multiplied by 100 % [32]. A gas chromatography (GC) of the Varian CP-3800 GC equipped with a flame ionization detector and a CP Sil 8 CB capillary column (25 m × 0.32 mm, 0.25 *μ*m film thickness) was used to determine the TPH and AC concentrations.

### Identification of bacterial isolates

A number of physical and biochemical tests were performed for the identification of bacterial isolates with the help of standard methods [33], such that: (1) morphology observation by Olympus-CH40 microscope, (2) lactose test, (3) indole test, (4) urea utilization test, (5) motility test, (6) H_2_S production test, (7) KCN growth test, (8) oxidase test, (9) gram test, (10) catalase test, (11) lysine dicarboxlaset test, (12) methyl red test, (13) Voges-Proskauer test, (14) citrate test, (15) pigment test, (16) dulcitol test, and (17) tartrate tests.

### Optimization of biological crude oil removal

The optimum conditions of biological CO removal were investigated by Taguchi method; therefore, five important factors of temperature, pH, initial CO concentration, hydraulic retention time (HRT), and nitrogen concentration were selected. Each factor was tested in three different conditions for which the temperatures of 20, 30 and 40 °C the pH of 6, 7 and 8, the initial CO concentrations of 0.05, 3 and 6 % (v/v), the HRTs of 5, 10 and 15 d, and the nitrogen concentrations of 0.2, 1 and 2 g/L were set up for the experiments. Note that NaNO_3_ was used as a source of nitrogen. The results obtained were analyzed using a dedicated computer program of the Qualitek software.

## Results and discussion

### Ability of the bacterial strains to degrade crude oil hydrocarbons

**Fig. 1:**
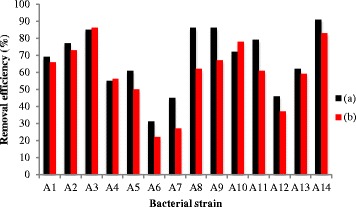
Removal efficiency of crude oil degraded by different bacterial strains; (**a**) TPH and (**b**) AC

**Fig 2:**
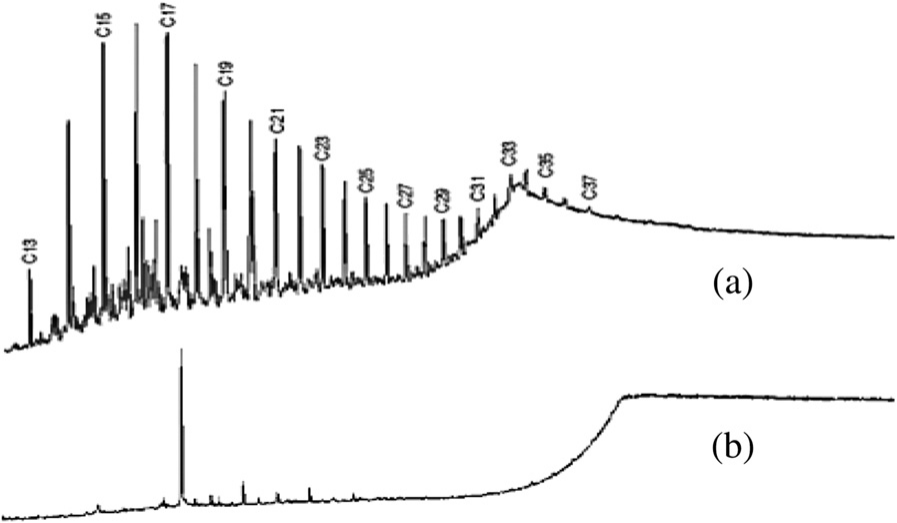
Results of GC analysis for the removal of TPH; (**a**) before degraded by *Pseudomonas aeruginosa* (**b**) after degraded by *Pseudomonas aeruginosa*

In this work, fourteen strains of oil-degrading bacteria were isolated from hydrocarbon-polluted samples coming from the areas of contaminated soils and effluents in Iran. The bacterial strains that could use CO as the sole carbon and energy source were noted as bacterial strains A1, A2, A3, A4, A5, A6, A7, A8, A9, A10, A11, A12, A13 and A14. All these bacterial strains are compared with each other in terms of their ability to remove TPH and AC from oily environment. In spite of the bacterial strain A3 has an ability to remove approximately 85 % of the TPH and approximately 86 % of the AC as shown in Fig. 1, the figure shows that after 10 days a higher efficiency in removing TPH (91 %) could only be the bacterial strain A14. The GC analysis (see Fig. 2) conformed that the use of bacterial strain A14 can remove approximately 91 % of the TPH. Therefore, the ability of bacterial strain A14 can remove TPH better than the strain A3. It seems that the ability of each bacterial strain to oxidize CO depends on direct cell-substrate contact and might be dependent on its ability to produce biosurfactants. The ability of bacterial strain A14 to remove AC would show slightly lower than that of bacterial strain A13. This may attribute to the cyclic structure of AC, which is more resistant to environmental degradation through chemical, biological and photolytic processes.

The growth of isolated bacteria in oily environment was investigated by inoculating the oil-degrading bacteria into a minimal culture medium (MCM) containing of 1 % (v/v) CO for 7 days. A 3 mL of bacterial strains was transferred from the MCM to a cuvette for the observation of cell growth identified by optimal optical density (OD_600_), measured by a DR5000 Spectrophotometer at a wavelength of 600 nm. The growth was categorized as low growth with OD_600_ in the range of 0.21-0.40, moderate growth in the range of 0.41-0.60, high growth in the range of 0.61-0.80, and excellent growth in the range of 0.81-1.00 [34]. The bacterial strain A14 has been identified as *Pseudomonas aeruginosa* [35]; the growth of such bacteria in oily environment can be categorized as high growth rate due to the OD_600_ value obtained of 0.661 should be in the range of 0.61-0.80. According the results of the optimization of biological CO removal, the optimum conditions of such a biological process are at a temperature of 30 °C, a pH of 7, an initial CO concentration of 3 % (v/v), a HRT of 5 d, and a nitrogen concentration of 1 g/L. The influence of each factor was ranked gradually from highest to lowest effect on the rate of CO degradation by *Pseudomonas aeruginosa*. The contributions of these factors are as follows: 27.36 % for pH, 19.33 % for nitrogen concentration, 10.31 % for initial CO concentration and 9.69 % for temperature. However, the influence of HRT on the CO removal by *Pseudomonas aeruginosa* would be nearly zero due to the experimental runs from 5 to 15 d have no effect on the increase in CO removal rate, suggesting that effective range of the HRT should be below than 5 d.

### Emulsification

**Fig. 3:**
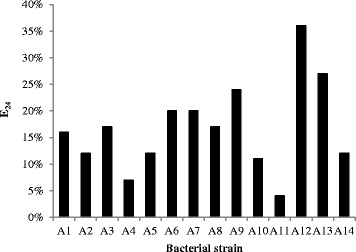
The value of E_24_ for different bacterial strains

Since only 0.02 % of CO is water soluble, there is a need for emulsification, which is necessary for improving the bioavailability of petroleum hydrocarbons to microorganisms by increasing the interfacial area between the water and the insoluble hydrocarbons [25, 36]. Biosurfactant-producing bacteria can consume CO as the sole carbon and energy source in various different forms including soluble CO, very small CO droplets and large CO droplets. As demonstrated by numerous emulsification studies over the past few decades [37, 38], the presence of biosurfactant decreases the equilibrium droplet size of emulsions. The emulsifying activity of biosurfactants produced by fourteen bacterial strains was checked after being allowed to settle for 24 h. Figure 3 shows that the highest values of E_24_ is 36 % for the bacterial strain A12, which has the ability to remove 46 % of the TPH and 37 % of the AC. However, the maximum values of TPH removal were 85, 86, 86 and 91 % achieved for the bacterial strains A3, A8, A9 and A14, respectively, while the corresponding E_24_ values were 17, 17, 24 and 12 %, respectively. In spite of all the bacterial strains can produce biosurfactant during growth on the petroleum hydrocarbons [39], the biosurfactant did not affect the rate of CO biodegradation. The use of *Pseudomonas aeruginosa* was able of removing high concentrations of TPH (91 %) and AC (83 %) without any biosurfactant addition.

### Kinetics of substrate utilization

**Fig 4:**
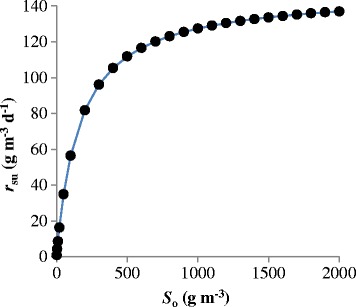
Estimation of *r*_*su*_ for different values of *S*_*0*_

Using Eq. (1) permits us to calculate *r*_su_ and thus can be plotted it with different *S*_o_ used for bacterial growth. The relationship between *r*_su_ and *S*_o_ (Fig. 4) shows that at low concentration of CO there is a steep increase in the rate of reaction with increasing CO concentration. However, at high concentration of CO, one of the approaches has been to slow down the rate of growth on a readily metabolizable substrate like TPH by the addition of different CO concentrations to obtain ready release of the biosurfactants into the CMBR. Due to the low aqueous solubility of CO, the ability of *Pseudomonas aeruginosa* to oxidize TPH depends on direct cell-substrate contact [40].

**Fig 5:**
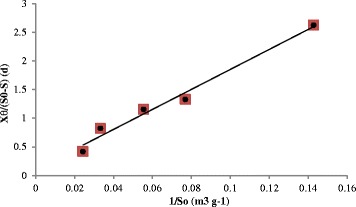
Graph of plotting $\frac{X \times \theta }{S_0-S}$ versus $\frac{1}{S_0}$ for the linear regression analysis

A plot (Fig. 5) of $\frac{X \times \theta }{S_0-S}$ versus $\frac{1}{S_0}$ as modeled in Eq. (2) gives a straight line with *a* equals 17.44 and *b* equals 0.1074. Therefore, the value of *k* is equal to 9.31 g of substrate g^−1^ of microorganism d^−1^ and the value of *K*_s_ is equal to 162.4 g m^−3^ of substrate. Correlation for all the parameters in equation is good (*R*^2^ = 0.9811, see Fig. 5). According to Eq. (1), the low *K*_s_ for CO means that high rate of substrate utilization can be achieved at the low concentration, while the high *K*_s_ for CO means that much higher concentration of microorganisms would be needed to increase the utilization rate of CO. An increase in the *k* value would increase the rate of CO utilization. The Monod kinetic parameters of *k* and *K*_s_ for petrochemical wastewater treatment have been reported in a previous study as 4.44 g of substrate g^−1^ of microorganism d^−1^ and 154.2 g m^−3^ of substrate, respectively [41]. However, the kinetics parameters of *k* and *K*_s_ for the activated sludge process for the removal of organic matter from domestic wastewater could be in the range of 2-10 g of COD g^−1^ of VSS d^−1^ with a typical values of 5 g of COD g^−1^ of VSS d^−1^ and in the range of 10-60 g m^−3^ of COD with a typical value of 40 g m^−3^ of COD, respectively [27]. The use of *Pseudomonas aeruginosa* to degrade CO has been shown to result in increased substrate utilization rate, as its value *k* of 9.31 g of substrate g^−1^ of microorganism d^−1^ could be far higher than the value *k* obtained for petrochemical wastewater treatment (4.44 g of substrate g^−1^ of microorganism d^−1^) and that for municipal wastewater treatment (5 g of COD g^−1^ of VSS d^−1^). The kinetics of substrate utilization would be the critically important factors that can be influenced by CO concentrations [42, 43]. With the value of *K*_s_ being around 162 g m^−3^ of substrate for oily wastewater treatment would work better if adding specific microorganisms at relatively high concentrations can accelerate start-up process by introducing *Pseudomonas aeruginosa*, which is capable of degrading CO in the oily environment.

### Microbial growth rate

**Fig 6:**
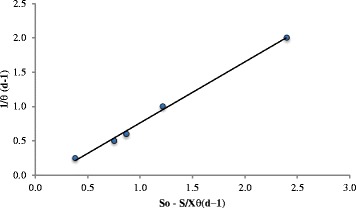
Graph of plotting of $\frac{1}{S_0}$ versus $\frac{S_0-S\ }{X \times \theta }$ for the linear regression analysis

A plot (Fig. 6) of $\frac{1}{\theta }$ versus $\frac{S_0-S\ }{X \times \theta }$ as modeled in Eq. (4) gives a straight line with *Y* equals 0.8896 g of biomass g^−1^of substrate and *k*_d_ equals 0.1284 g of microorganisms g^−1^ of biomass d^−1^. Correlation for all the parameters in equation is very good (*R*^2^ = 0.9961, see Fig. 6). The microbial parameters of *Y* and *k*_d_ for the activated sludge process for the removal of organic matter from domestic wastewater are in the range of 0.3-0.6 mg of VSS g^−1^ of COD with a typical value of 0.4 mg of VSS g^−1^ of COD and in the range of 0.06-0.15 g of VSS g^−1^ of VSS d^−1^ with a typical value of 0.10 g of VSS g^−1^ of VSS d^−1^, respectively [27]. Using Eq. (5) permits us to calculate the value of *μ*_max_, which is equal to 8.28 g of biomass (new cells) g^−1^ of microorganisms (existing cells) d^−1^. It seems that the production of new cells of using CO as the source of carbon and energy can exceed 2^3^ of the existing cells per day. The ability of *Pseudomonas aeruginosa* to degrade CO can be a frontrunner in creating a biological process for advanced oily wastewater treatment that can accelerate the development of sustainable biomass production in a CMBR, required the standardization of key technology and design parameters and processes.

## Conclusions

This study used the *Pseudomonas aeruginosa* to remove CO from oily environment applied to a CMBR. The removal efficiencies of TPH and AC were verified as high as 91 and 83 %, respectively, over a process period of 10 days. In spite of using the *Pseudomonas aeruginosa* to degrade CO can produce biosurfactant during growth on the petroleum hydrocarbons, such a bioproduct did not affect the CO degradation rate. The use of *Pseudomonas aeruginosa* to degrade CO has been shown to result in increased substrate utilization rate. The kinetics of substrate utilization and bacterial growth of using CO as the sole carbon and energy source were determined to contribute to improving the biological process for advanced oily wastewater treatment.
